# Variants in the new E1ʹ cryptic exon of the *VHL* gene associated with congenital erythrocytosis—Description of three cases

**DOI:** 10.1002/jha2.490

**Published:** 2022-07-01

**Authors:** Catarina Dantas Rodrigues, Rita Pombal, Janet Pereira, Luís Relvas, Elizabete Cunha, José Carlos Almeida, Tabita Maia, Helena Silva, Celeste Bento

**Affiliations:** ^1^ Serviço de Hematologia Centro Hospitalar de Tondela Viseu Viseu Portugal; ^2^ Serviço de Imunohemoterapia Centro Hospitalar de Vila Nova de Gaia/Espinho Gaia Portugal; ^3^ Eritropatologia e Metabolismo do Ferro, Serviço de Hematologia Clínica Centro Hospitalar Universitário de Coimbra Coimbra Portugal; ^4^ CIAS, Centro de Investigação em Antropologia e Saúde Universidade de Coimbra Coimbra Portugal

**Keywords:** congenital erythrocytosis, erythrocytosis, polycythaemia, VHL, von Hippel–Lindau

## Abstract

Congenital erythrocytosis (CE) represents a rare and heterogeneous group of hereditary disorders. The molecular basis of *VHL* gene mutations related to CE. Recently, Lenglet et al. reported a discovery of a novel cryptic exon in the *VHL* gene. Mutations in the first intronic region resulting in the creation of a cryptic exon termed E1ʹ were found in seven families with CE and one family with VHL disease. We report three patients with prolonged CE with the aetiology being clarified several years later by sequencing of intronic region 1 of the *VHL* gene. This work addresses the first cases reported at the clinical level of VHL‐associated CE due to the E1ʹ cryptic exon.

## 1 BACKGROUND

Congenital erythrocytosis (CE) represents a rare and heterogeneous group of hereditary disorders leading to increased red blood cell production, characterised by an increase in haemoglobin (Hb) concentration and haematocrit (Hct) level (adjusted to age, sex and altitude). Genetic changes affecting the regulatory pathway of erythropoiesis have been described in patients with CE, namely, due to mutations in the *EPAS1*, *EGLN1* and *VHL* genes [1, 2]. The von Hippel–Lindau tumour suppressor gene (*VHL*) encodes a multifunctional protein (pVHL). The VHL transcript contains three spliced exons (E1, E2 and E3) that encode pVHL213 (or pVHL30) and pVHL19 (pVHL160). Both are involved in the regulation of the cellular oxygen‐sensing pathway, specifically in the ubiquitination and degradation of hypoxia‐inducible factor (HIF), a transcription factor that plays a central role in the regulation of gene expression by oxygen. *VHL* mediates tumour invasion and metastasis by regulating HIF protein expression, and *VHL* mutations predispose patients to several angiogenic tumours (familiar VHL tumour syndrome) and familial erythrocytosis 2 (ECYT2) [2, 3]. The molecular basis of *VHL* gene mutations related to CE was first described in 2002 in the autonomous Russian Republic of Chuvashia. Chuvash polycythaemia arises from a homozygous *VHL*:c.598C>T p.Arg200Trp (R200W) mutation. Since this discovery, additional *VHL* mutations have been identified in patients with CE, with a recessive transmission. However, in some patients with CE, only one *VHL* mutation has been identified [4, 5]. Recently, Lenglet et al. reported a discovery of a novel cryptic exon in the *VHL* gene. Mutations in the first intronic region resulting in the creation of a cryptic exon termed E1ʹ were found in seven families with CE and one family with VHL disease. One patient (described below) had erythrocytosis of unknown origin, and a c.340+816A>C variant was identified in the homozygous state, with both parents being healthy and heterozygous for this mutation.

To date, no other cases of VHL E1ʹ mutations associated with CE have been reported. Here, we describe the clinical manifestations of three patients, two new and one already reported by Lenglet et al. [2], with lifelong erythrocytosis with an obscured aetiology clarified several years later by sequencing of intronic region 1 of the *VHL* gene.

## 2 CASES PRESENTATION

### 2.1 Case 1

A 5‐year‐old child complaining of recurrent frontal headaches and aquagenic pruritus. Physical examination was normal, except for plethoric facies. Blood count analysis showed increased Hb of 190 g/L and Hct of 58%, with normal white blood cell and platelet counts and an increased Erythropoietin (EPO) level of 35 mUI/ml (normal range 4.3–29.0 mUI/ml). Family history was negative, with no consanguinity. He did not have problem in sleeping or breathing. Abdominal ultrasound, electrocardiogram, echocardiography, pulmonary function tests, chest radiography and brain magnetic resonance imaging were normal. Hb electrophoresis did not reveal any abnormal Hb variant. Cortisol and testosterone levels were appropriate for his age. Bone marrow biopsies (at 7 and 8 years old) only showed erythroid hyperplasia. Genetic testing was negative for PV *JAK2* V617F mutations and for exonic mutations in all the genes described as associated with CE. Phlebotomies on demand were started to ameliorate symptoms.

### 2.2 Case 2

Forty‐six‐year‐old male with a history of erythrocytosis since age of 9 years. At the time he complained of recurrent epistaxis, with Hb of 165 g/L, Hct of 49%–57% and an increased EPO level of 135 mUI/ml (normal range 4.3–29.0 mUI/ml). Similar to the first case described, secondary causes were excluded, such as cardiac or pulmonary diseases. The presence of high‐affinity Hb variants or mutations involving the genes associated with CE and oxygen‐sensing pathway enzymes were also excluded. His parents were blood relatives (second cousins) and had normal blood counts, as well as his brother and two daughters. He is now asymptomatic but still needs phlebotomies every 6 months to remain stable.

### 2.3 Case 3

Eighty‐six‐year‐old male with a history of erythrocytosis known at least from 2011 when he had a stroke. Secondary causes were excluded, and genetic testing was negative. Family history was not available. Recently, he was reassessed and presented with Hb of 190 g/L, Hct of 60% and increased EPO level of 45.4 mUI/ml (normal range 4.3–29.0 mUI/ml). He has been doing phlebotomies on demand, as well as warfarin due to the history of stroke and multiple cardiovascular risks.

**FIGURE 1:**
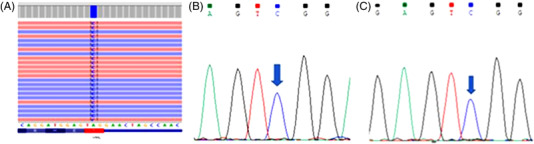
Identification of VHL deep‐intronic variant VHL:c.340+616A>C in homozygous state. (A) NGS gene panel, patient 3; (B) Sanger sequencing, patient 1; (C) Sanger sequencing, patient 2. NGS, next‐generation sequencing

Sequencing of the intronic region 1 E1ʹ of *VHL* led to the identification of the variant VHL:c.340+816A>C in the homozygous state in all three patients (Figure 1).

## 3 CONCLUSION

The *VHL* gene plays a pivotal role in the oxygen‐sensing pathway and subsequently in erythropoiesis.

Lenglet et al. reported that mutations in the first intronic region resulting in the creation of a cryptic exon termed E1ʹ were found in seven families with CE and one family with VHL disease. These mutations led to an abnormal *VHL* mRNA with the insertion of the E1ʹ in the transcript and to mRNA degradation and failure to protein expression. They did not have an impact on the coding sequence but influenced VHL splicing, downregulating VHL expression and leading to an impairment of HIF degradation and *EPO* upregulation. This study led to the identification of E1ʹ heterozygous mutations in the second allele of six families with CE previously associated with a heterozygous mutation in *VHL*. This, in addition to the identification of E1ʹ expressed in healthy tissues, helped to confirm that E1ʹ mutations are associated with CE.^2^ This VHL:c.340+816A>C intronic mutation has been detected in three patients with clinical symptoms of erythrocytosis, which had been of unknown aetiology until this moment.

Patients with presumed CE and VHL disease whose genetic testing excluded mutations in the coding sequence should be screened for changes within VHL intronic sequences that affect exon splicing. The detection of these molecular changes has clinical implications, since phlebotomy in VHL‐related erythrocytosis may increase the risk and severity of pulmonary hypertension, and regular follow‐up is necessary to assess the risk of thromboembolic complications, pulmonary hypertension, cardiovascular disease and VHL‐related tumours.

More studies are required to better understand genotype–phenotype correlations and the most suitable therapeutic approach.

To the best of our knowledge, these are the first cases reported at the clinical level of VHL‐associated CE due to the E1ʹ cryptic exon.

## CONFLICT OF INTEREST

The authors declare that they have no conflicts of interest.

## FUNDING INFORMATION

The authors received no financial support for the research, authorship, and/or publication of this article.

## ETHICS STATEMENT

Ethics approval for this study was obtained from Local Regional Ethics Boards.

## PATIENT CONSENT STATEMENT

Informed consent was obtained.

## AUTHOR CONTRIBUTIONS

Catarina Dantas Rodrigues took the lead in writing the manuscript with support from all authors. Rita Pombal wrote one of the cases and provided critical feedback. Luís Relvas, Janet Pereira and Elizabete Cunha are laboratory technicians who performed the techniques. João Carlos Almeida, Tabita Maia and Helena Silva are physicians of the study subjects. Celeste Bento worked as study coordinator and head of laboratory.

## Data Availability

The data that support the findings of this study are available on request from the corresponding author. The data are not publicly available due to privacy or ethical restrictions.
